# Green tea catechins alleviate autoimmune symptoms and visual impairment in a murine model for human chronic intraocular inflammation by inhibiting Th17-associated pro-inflammatory gene expression

**DOI:** 10.1038/s41598-019-38868-1

**Published:** 2019-02-19

**Authors:** Jian Li, Yolanda Wong Ying Yip, Jialin Ren, Wing Ki Hui, Jing Na He, Qiu Xiao Yu, Kai On Chu, Tsz Kin Ng, Sun On Chan, Chi Pui Pang, Wai Kit Chu

**Affiliations:** 1 Department of Ophthalmology & Visual Sciences, The Chinese University of Hong Kong, Hong Kong, Hong Kong; 2 Department of Ophthalmology, Affiliated Hangzhou First People’s Hospital, Zhejiang University School of Medicine, Hangzhou, China; 3 School of Biomedical Sciences, The Chinese University of Hong Kong, Hong Kong, Hong Kong; 4 Bachelor of Medicine and Bachelor of Surgery Programme, The Chinese University of Hong Kong, Hong Kong, Hong Kong

## Abstract

Autoimmune uveitis is a sight-threatening disease mainly caused by dysregulation of immunity. We investigated the therapeutic effects of green tea extract (GTE) and its major component, epigallocatechin-3-gallate (EGCG), on a murine model of experimental autoimmune uveoretinitis (EAU). Oral administration of GTE, EGCG, dexamethasone, or water, which started 5 days before the induction, was fed every two days to each group. On day 21 post induction, the eyes were examined by confocal scanning laser ophthalmoscopy, optical coherence tomography (OCT), fundus fluorescein angiography (FFA) and electroretinography (ERG) prior to sacrificing the animals for histological assessments and gene expression studies. Retinal-choroidal thicknesses (RCT) and major retinal vessel diameter were measured on OCT sections and FFA images, respectively. Comparing to water-treated EAU animals, GTE attenuated uveitis clinical manifestations, RCT increase (1.100 ± 0.013 times vs 1.005 ± 0.012 times, *P* < 0.001), retinal vessel dilation (308.9 ± 6.189 units vs 240.8 units, *P* < 0.001), ERG amplitudes attenuation, histopathological ocular damages, and splenomegaly in EAU mice. The therapeutic effects of GTE were dose dependent and were comparable to dexamethasone. EGCG, a major active constituent of GTE, partially alleviated uveitic phenotypes including recovering visual function. Th-17 associated pro-inflammatory gene [interleukin 1 beta (*IL-1β)*, *IL-6*, *IL-17A*, and tumor necrosis factor alpha *(TNF-α)*] expressions were down regulated by GTE and EGCG treatments, which showed no detectable morphological defects in liver and kidney in non-induced and EAU mice. Our findings suggest that GTE consumption can serve as a potent therapeutic agent as well as a food supplement for developing alternative treatments against autoimmune uveitis.

## Introduction

Uveitis with complex manifestations of intraocular inflammation is one of the leading causes of blindness^1^. More than 150 disorders are reported to be associated with intraocular inflammation. Some are caused by infectious agents and rehabilitated by specific antimicrobial therapies with or without corticosteroids. Others are of putative autoimmune nature as the underlying infectious trigger could not be identified. Thus, for the chronic autoimmune uveitis, corticosteroids are the mainstay of therapy. However, there are some uveitis patients who fail to respond to the current treatments. In addition, the long-term treatments may have several intraocular and systemic side effects such as high intraocular pressure and nephrotoxicity. In cases of severe sight-threatening uveitis, surgeries are needed to prevent the secondary visual impairments^2^. Therefore, alternative therapies are warranted for the autoimmune intraocular inflammation.

The disorders are associated with autoimmune responses to retinal proteins. Currently, numerous important findings of basic immunological mechanisms and novel therapies in human autoimmune uveitis have been revealed by animal studies based on a mouse model of experimental autoimmune uveoretinitis (EAU) targeting human inter-photoreceptor retinoid binding protein (hIRBP)^3–5^. Previously, to objectively evaluate the therapeutic effects of anti-inflammatory agents in EAU mice, we developed a novel evaluation system consists of three quantitative indicators, retinal-choroidal thickness (RCT), major retinal vessel diameter and electroretinography (ERG) amplitudes^6^.

Green tea extract (GTE) is isolated from the non-fermented leaves of *Camellia Sinensis*. The most abundant constituent of green tea polyphenols is (−)-epigallocatechin gallate (EGCG)^7^, which constitutes 70% of GTE in terms of weight. EGCG has been shown to inhibit the human immunodeficiency virus (HIV) infection^8^ and multidrug-resistant *Staphylococcus aureus* infections^9^. Our previous studies revealed the protective effects of GTE in ocular diseases such as acute infectious inflammation in endotoxin-induced uveitis (EIU) and sodium iodate-induced oxidative retinal degeneration in rats^10,11^.

However, the effects of GTE and EGCG in intraocular autoimmune inflammation are still unknown. The present study demonstrated the therapeutic effects of oral intake of GTE and EGCG in a mouse model of EAU, using both *in vivo* and *in vitro* techniques to evaluate the severity of EAU.

## Results

### GTE attenuates clinical manifestations and histopathological ocular damage in EAU eyes

**Figure 1:**
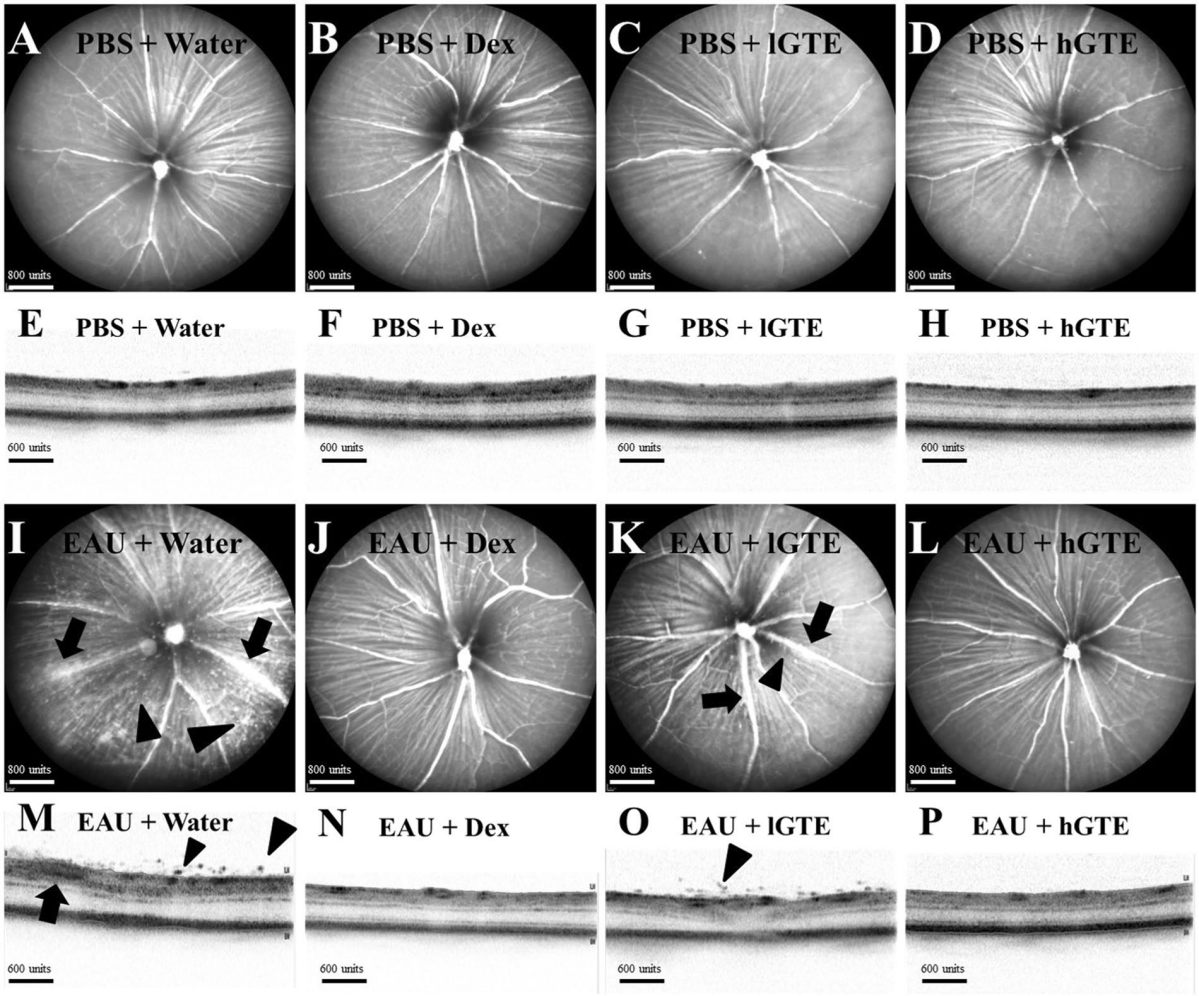
Clinical manifestation of inflammation observed by cSLO and SD-OCT. No detectable changes were observed in cSLO images **(A**–**D)** and SD-OCT images **(E–H)** of non-induced animals treated with water, dexamethasone, lGTE, or hGTE. cSLO images **(I**–**L)** and SD-OCT images **(M**–**P)** of EAU animals administrated with water, dexamethasone, lGTE, or hGTE are shown. Infiltrating cells (*arrow heads*) and vasculitis (*arrows*) were observed, indicating inflammation was induced after immunizing the animals **(I**,**M)**. Sporadic infiltrating cells (*arrow heads*) and mild vasculitis (*arrows*) were observed in EAU animals treated with lGTE **(K**,**O)**. Inflammatory responses were subsided in mice treated with dexamethasone **(J**,**N)** or hGTE **(L**,**P)**.

As indicated in Fig. 1, *in vivo* monitoring showed intraocular inflammation by EAU induction on day 21 postimmunization (d21pi). Comparing with the mild inflammation in 137.5 mg/kg of GTE (lower dose of GTE, lGTE), 275 mg/kg of GTE (higher dose of GTE, hGTE) treated animals and Dex-treated EAU groups showed less severe EAU. Clinical scores of EAU (Fig. S1) showed significant reductions in hGTE (0.125 ± 0.065, *P* < 0.01) and Dex-treated EAU groups (0.292 ± 0.168, *P* < 0.05) when compared with water-treated EAU animals (0.917 ± 0.267).

**Figure 2:**
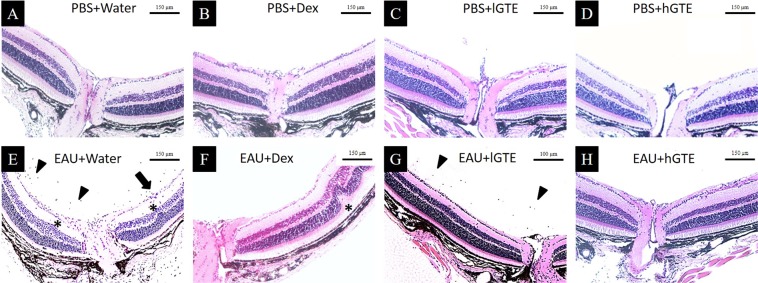
Histological observations of eyeballs at d21pi. H&E sections showed normal structures of posterior segment of the eyeballs in non-induced animals with different treatments **(A**–**D)**. Infiltrating cells (*arrow heads*), vasculitis (*arrow*), and retinal folds (*asterisks*) were observed in EAU + Water group (**E**). Retinal fold (*asterisk*) was found in EAU + Dex group (**F**). Mild cell infiltration (*arrow heads*) was observed in EAU + lGTE group **(G**). No detectable change in posterior segment was observed in EAU animals treated with hGTE (**H**).

Histopathology (Fig. 2) also detected inflammatory damages in water-treated EAU mice. Inflammation was not observed in hGTE treated EAU group. However, mild vitritis and retinal folds were detected in lGTE and Dex treated EAU animals.

### GTE alleviates retinal-choroidal edema, retinal vasodilation and visual impairment in EAU mice

**Figure 3:**
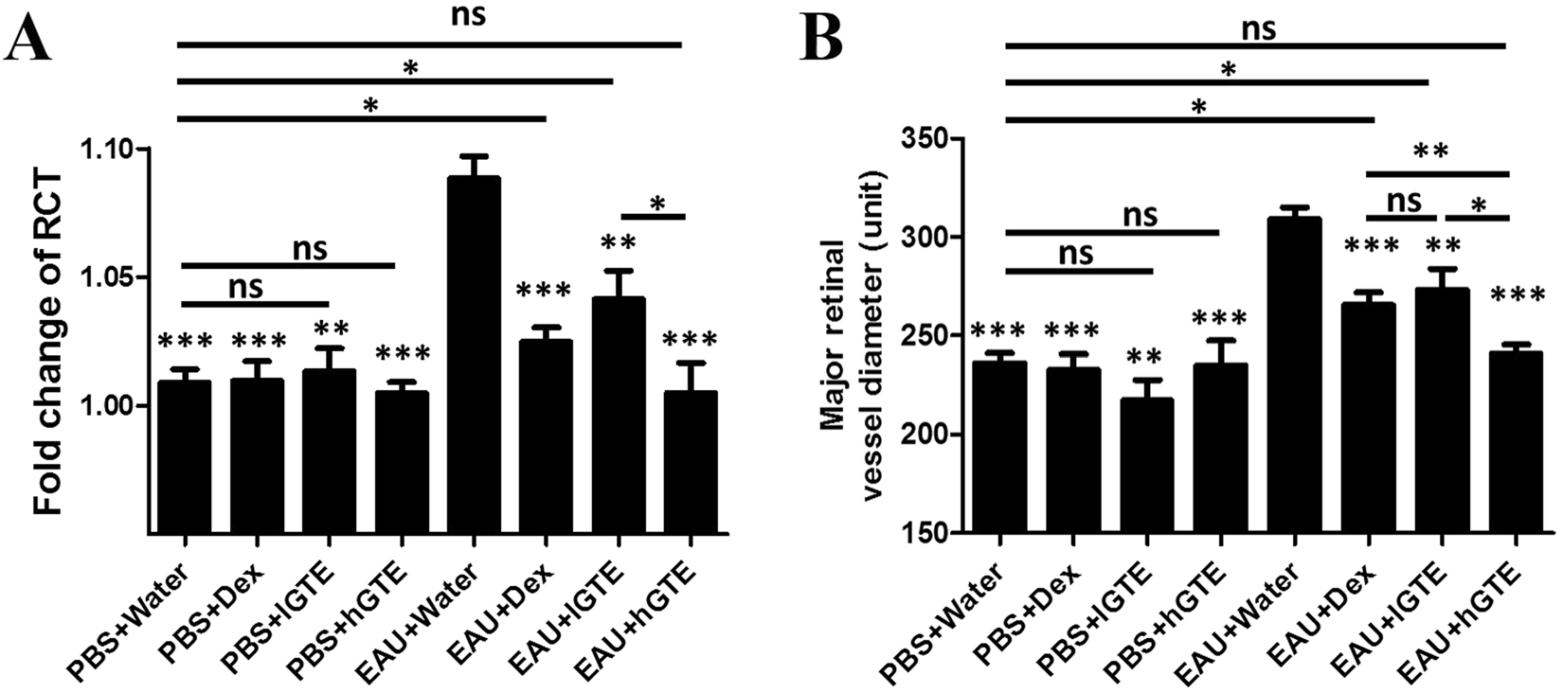
The fold change of RCT (**A**) and the major retinal vessel diameter (**B**) assessments. The fold changes of RCT and major retinal vessel diameters were reduced significantly after Dex, lGTE, and hGTE treatment in EAU mice. The reductions between different doses of GTE were statistically significant in EAU mice. However, the reduced fold changes of RCT and major retinal vessel diameters in Dex and lGTE treatment groups, but not hGTE-treated group, were still significantly higher than PBS + Water group. There were no differences between PBS induction groups treated with water, lGTE, and hGTE. The asterisk marked above each bar represents the statistical significance of comparison between EAU + Water group and the corresponding group. Data are also presented as mean ± SEM and analyzed by Mann–Whitney *U* test (*P < 0.05, **P < 0.01, ***P < 0.001, ns = no significance).

EAU induction significantly increased the RCT during EAU (*P* < 0.001). We calculated the fold change of RCT by dividing d21pi RCT with baseline RCT. The fold changes of RCT in EAU mice with lGTE, hGTE and Dex treatments were all significantly alleviated when compared with water-treated EAU mice (*P* < 0.01). Nonetheless, the fold change of RCT in lGTE treatment group was significantly higher than that in hGTE-treated mice (*P* < 0.05) (Fig. 3A).

Major retinal vessel diameter was significantly increased by EAU induction (308.90 ± 6.19 units, *P* < 0.001). The vasodilation caused by EAU was decreased significantly by lGTE (273.20 ± 10.79 units, *P* < 0.05), hGTE (240.80 ± 4.66 units, *P* < 0.001) or Dex (265.50 ± 6.42 units, *P* < 0.001) treatments. hGTE further showed smaller vessel diameter than Dex (*P* < 0.01) and lGTE treatments (*P* < 0.05). Major retinal vessel diameters in phosphate buffer saline (PBS) mock induced animals treated with water, lGTE or hGTE were comparable (*P* > 0.05) (Fig. 3B).

Intra-group comparisons of scotopic (dark-adapted) (Fig. S2) and photopic (light-adapted) (Fig. S3) ERG amplitudes between d21pi and baseline revealed significant impairment of visual function by EAU (*P* < 0.01). ERG amplitudes in Dex and hGTE treated EAU animals were maintained during the experiment (*P* > 0.05).

**Figure 4:**
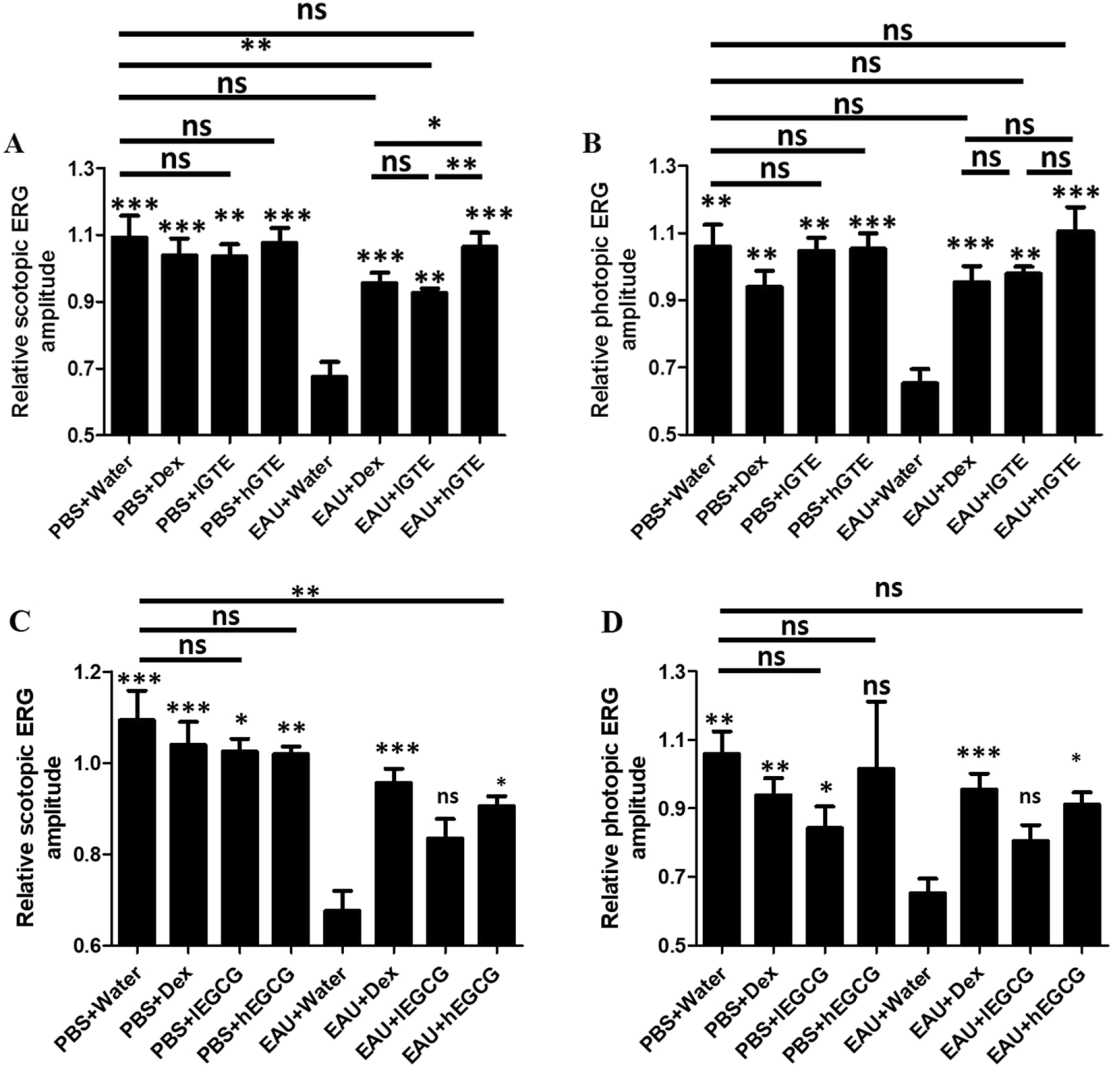
Inter-group comparisons of scotopic (**A**,**C**) and photopic (**B**,**D**) ERG amplitudes. Relative ERG amplitude was calculated as amplitude at d21pi divided by that at baseline. The asterisk marked above each bar represents the statistical significance of comparison between EAU + Water group and the corresponding group. Data are also presented as mean ± SEM and analyzed by Mann–Whitney *U* test (*P < 0.05, **P < 0.01, ***P < 0.001, ns = no significance).

Inter-group differences were evaluated by comparing scotopic (Fig. 4A) and photopic ERG amplitudes (Fig. 4B) under their highest stimuli in this study. Relative ERG amplitude was calculated as amplitude d21pi divided by that on baseline. EAU significantly caused reduction of scotopic (*P* < 0.001) and photopic ERG (*P* < 0.01). lGTE, hGTE and Dex treatments alleviated the attenuations of visual function caused by EAU (*P* < 0.01).

### EGCG could not attenuate clinical manifestations, retinal-choroidal edema and retinal vasodilation in EAU eyes

Inflammation appeared in both doses of EGCG treated EAU mice (Fig. S4). Clinical scores of EAU showed no significant differences between EGCG treated EAU groups and water-treated EAU mice (Fig. S5). A superior treatment effect of hGTE was observed by directly comparing EGCG (low and high doses) and GTE (low and high doses) using Mann-Whitney test (*P* < 0.05 in all three comparisons of hGTE group against lGTE, hEGCG and lEGCG groups). Multiple comparison of EGCG (low and high doses) and GTE (low and high doses) by Kruskal-Wallis test showed statistical significance (*P* = 0.0135).

Fold changes of RCT in both lEGCG (1.06 ± 0.01) and hEGCG (1.07 ± 0.04) treatments in EAU groups had no significant differences from water-treated EAU animals (EAU + Water group) (1.10 ± 0.01, *P* > 0.05). (Fig. S6A). By comparing EGCG (low and high doses) and GTE (low and high doses), we found statistical significance in Kruskal-Wallis test (*P* = 0.0384). Mann-Whitney test showed that hGTE was more effective in attenuating retinal-choroidal edema than lEGCG (*P* = 0.0169).

The major retinal vessel diameter increased by EAU induction (308.90 ± 6.19 units) was decreased by lEGCG (292.20 ± 14.43 units) and hEGCG (290.80 ± 9.32 units) treatments. However, the differences were not statistically significant (*P* > 0.05) (Fig. S6B). Multiple comparison among EGCG (low and high doses) and GTE (low and high doses) treatment groups by Kruskal-Wallis test showed significant difference (*P* = 0.0011). Direct comparisons of hGTE treatment against hEGCG or lEGCG showed significant stronger therapeutic effects by using Mann-Whitney test (*P* = 0.0023 and *P* = 0.017 respectively).

### High dose EGCG alleviates visual impairment in EAU mice

hEGCG treatment group showed no significant reductions of scotopic (Fig. S7) and photopic (Fig. S8) ERG amplitudes in the intra-group comparisons (*P* > 0.05).

In the inter-group comparisons (Fig. 4C,D), hEGCG treatment significantly alleviated the attenuations of visual function caused by EAU (*P* < 0.05). The alleviated photopic ERG amplitude by hEGCG in EAU animals was comparable with PBS + Water animals (*P* > 0.05). However, the scotopic ERG in hEGCG group was still significantly lower than that in water-treated PBS animals (*P* < 0.01).

### GTE and EGCG alleviate the EAU associated changes of spleen but do not affect the structures of liver and kidney

Splenomegaly is associated with severity of autoimmune disorders in both animal studies^12^ and clinical investigations^13,14^. Both spleen weight and size were significantly elevated by EAU (*P* < 0.05). Spleen weight and size in lGTE and hGTE treatment groups were significantly lower than that in EAU + Water group (*P* < 0.05) (Fig. S9). The spleen weight and size in two doses of EGCG treatment groups were significantly lower than that in EAU + Water group (*P* < 0.05), except for the spleen size in lEGCG-treated animals (*P* > 0.05) (Fig. S10).

Observed from histological verification, GTE treatment groups showed less accumulation of infiltrating cells in spleen comparing with water-treated EAU mice (Fig. S11). However, no detectable defects of liver and kidney were found in all groups (Figs S12 and S13).

### GTE and EGCG downregulate Th17-associated pro-inflammatory gene expressions

**Figure 5:**
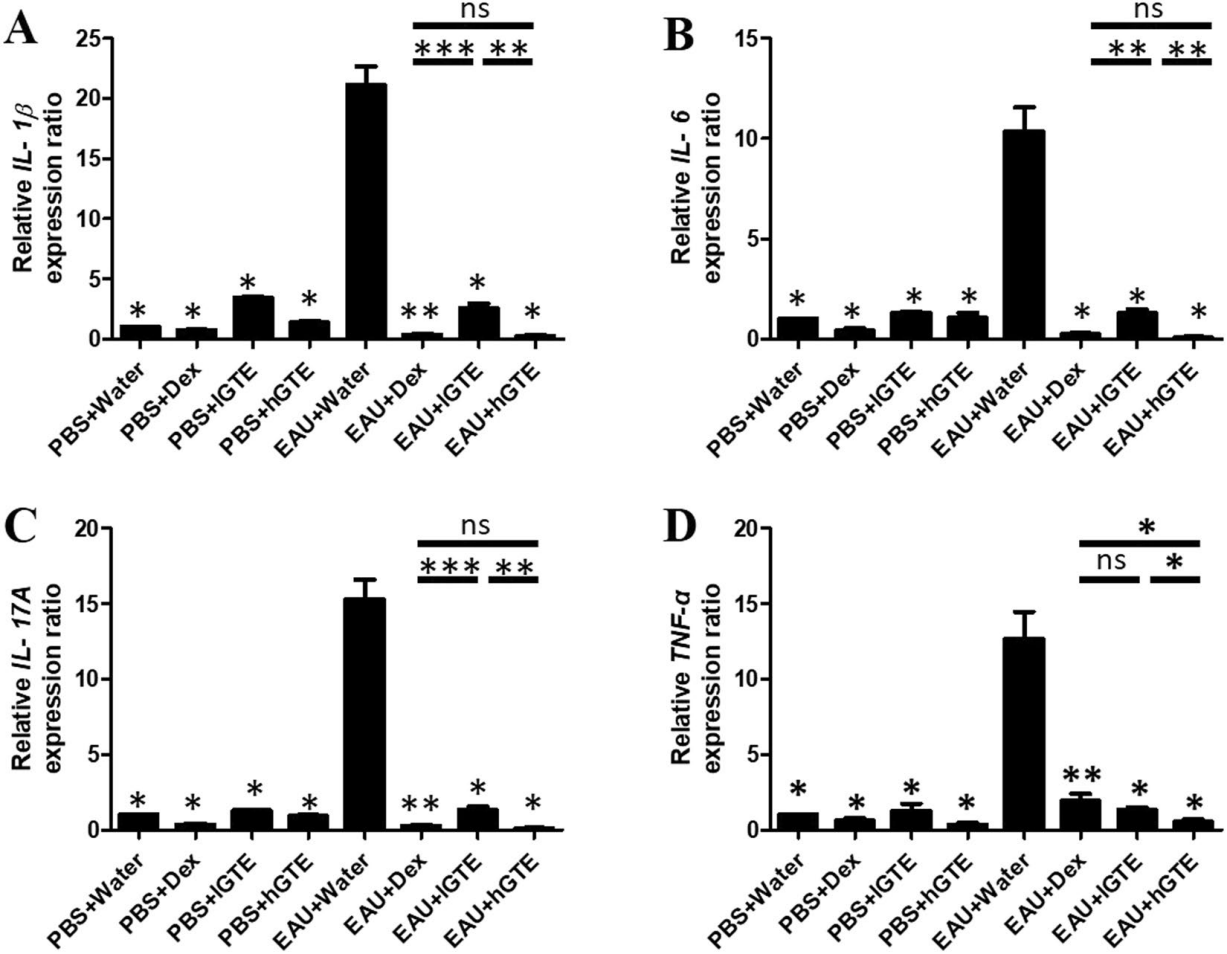
Effects of GTE on *IL-1β*, *IL-6*, *IL-17A*, and *TNF-α* mRNA expression in EAU retina. The asterisk marked above each bar represents the statistical significance of comparison between EAU + Water group and the corresponding group. Data are shown as mean ± SEM and analyzed using Mann-Whitney *U* test (*P < 0.05, **P < 0.01, ***P < 0.001, ns = no significance).

The expressions of pro-inflammatory gene interleukin 1 beta (*IL-1β)*, *IL-6*, *IL-17A*, and tumor necrosis factor alpha *(TNF-α)* were at basal levels in the PBS controls, but increased significantly after EAU induction. The elevated levels of *IL-1β*, *IL-6*, *IL-17A*, and *TNF-α* were significantly downregulated after treatment of lGTE, hGTE, lEGCG, hEGCG and Dex (*P* < 0.05) when compared to the water-treated EAU group (Figs 5 and S14). hGTE treatment showed more effective suppression of *TNF-α* than lGTE and Dex (*P* < 0.05). For *IL-1β*, *IL-6*, and *IL-17A* expressions, hGTE and Dex treatments showed comparable suppression effects which were stronger than lGTE treatment (*P* < 0.05).

## Discussion

To develop alternative therapies for patients with autoimmune uveitis, we investigated the anti-inflammatory effects of GTE against EAU mouse model. With GTE acting as a food supplement, we pre-treated the animals once per two days which started from 5 days before EAU induction and lasted to the endpoint of experiment. Our results suggest for the first time that GTE is a potent anti-inflammatory agent for autoimmune ocular inflammation.

By overviewing the effects of EGCG and comparing them with GTE, we found that the equivalent dosages of EGCG alone cannot alleviate EAU as effectively as GTE. Hence, besides EGCG, other catechins and components in GTE may also play roles in EAU alleviation. The present attempts were able to reveal a very limited understanding of GTE and EGCG effects. In future studies, different treatment intervals (other than once every 2 days) and the influence of different schedules on inflammatory process could be evaluated. Further studies are also proposed to investigate the different effects of various components of GTE. To determine the active duration of the GTE ingredients in mice, blood levels of GTE ingredients at periodic intervals should be documented in the future. It is also worth considering that if pure EGCG is more difficult to be absorbed via the gut, or if EGCG has a shorter half-life in bloodstream when it is not conjugated with other components in GTE. Future pharmacological studies may provide more information to these hypotheses.

In the IRBP/CFA-EAU model, CD4+ Th17 (IL-17-producing) cells dominantly participate in the inflammation^15^. IL-1β and IL-6 are critical in determining the lineage choice of differentiating Th17 cells. IL-17A and TNF-α are the hallmark pathogenic cytokines produced by Th17 cells^16^. IL-6 also activates CD8+ T cells and inhibits differentiation of regulatory T (Treg) cells^17,18^. Administration of anti-IL-1β or anti-IL-6 antibody has been shown to attenuate EAU in mice^19,20^ and humans^21^. Based on our observations, GTE and EGCG effectively downregulated the *IL-1β*, *IL-6*, *IL-17A*, and *TNF-α* expression in EAU, suggesting that GTE and EGCG inhibit EAU by targeting the Th17-associated pro-inflammatory gene expression.

In this study, animals were treated with 1 mg/kg of dexamethasone as a positive control. GTE treatment was comparable with dexamethasone administration for most of the observations, or even better in remissions of clinical inflammatory manifestations, vasodilation, and *TNF-α* expression. EGCG showed less effective alleviation of EAU comparing with dexamethasone, except for maintaining ERG amplitudes and suppressing some of the pro-inflammatory gene expressions.

Safety issues are the main concern in treating uveitis clinically. We evaluated the differences of all parameters measured in our present study among the PBS mock induced control groups and EAU groups treated with water, GTE and EGCG. No significant changes were found among control groups treated with water and different doses of GTE and EGCG, implying a well tolerance of GTE and EGCG in healthy individuals. Additionally, histological assessments of liver and kidney after these treatments also showed no detectable changes in all groups. One of the most important problems with steroids is the side effects they caused. However, we did not observe any histological evidence of toxicity in the liver and kidney in the Dex-treated mice. It will be worthwhile to comprehensively assess the safety of higher doses GTE, EGCG and Dex treatment for murine and human uveitis in future studies. Further studies were warranted to develop clinical trials on GTE and EGCG treatments for uveitis patients. *In vivo* measurements employed in the current study could be directly translated to patients, as they are widely used in clinical practice. However, the treatment dosages in animals should definitely not be directly copied to humans without adjustment, as the metabolisms in human are very different from animals. Since human data are limited, we could only predict future administration of GTE in humans referring to the existing animal studies. The equivalently translating doses for human use could be calculated by using the isocaloric method^22^. Another concern was raised as the amount of catechins and GTE used in this study are not physiological. These doses are much higher than the reasonable intake of green tea. In real life, much less green tea would have been consumed comparing to the highly concentrated dosages in our experiments. However, after testing two different doses of GTE and EGCG, we found a dose-dependent effect of the catechins. The higher dose showed more potent alleviation of inflammation in EAU. Although it is not realistic to achieve the effective dose of GTE by drinking green tea, it would be possible to achieve this concentration by taking purified GTE capsules.

## Conclusion

In conclusion, our *in vivo* and *in vitro* assessments showed that oral administration of GTE alleviates autoimmune inflammation in perspectives of morphology and visual function by inhibiting Th17-associated pro-inflammatory gene expressions. Our findings suggest that GTE consumption can serve as a potent therapeutic agent as well as a food supplement for treatment of intraocular autoimmune inflammation.

## Materials and Methods

### Animals

C57BL/6 J mice were obtained from the Laboratory Animal Service Center of The Chinese University of Hong Kong (CUHK). Animals were housed in standard condition, maintained at 22 ± 1 °C in 40 ± 10% humidity, and 12/12 hour light-dark cycles with free access to food and water. All experiments were conducted according to the Association for Research in Vision and Ophthalmology (ARVO) statement for the use of Animals in Ophthalmic and Vision Research. Ethics approval for this study was obtained from the Animal Ethics Committee of CUHK.

### Induction of EAU

EAU induction was performed as described previously^23,24^. In brief, 400 μg hIRBP peptide 1-20 (GPTHLFQPSLVLDMAKVLLD; AnaSpec Inc., USA) was emulsified with an equal volume of complete Freund’s adjuvant (CFA) containing 4.5 mg/mL *Mycobacterium tuberculosis* H37RA (Difco, USA). A total of 200μL emulsion was injected subcutaneously at the base of the tail (100 μL) and both thighs (50 μL each). Pertussis toxin (PTX) (0.1 μg in 100 μL; Sigma-Aldrich Corp., USA) was injected intraperitoneally immediately after immunization. Control animals were injected with equal volume of sterile PBS.

### GTE and EGCG treatments

Commercially available decaffeinated GTE, Theaphenon E, was kindly provided by Dr. Yukihiko Hara (Shimane University Faculty of Medicine, Japan), which contains epigallocatechin gallate (EGCG, 70%w/w), epigallate catechins (EGC, 4.6%w/w), epicatechin (EC, 3.88%w/w) and epicatechin gallate (ECG, 6.9%w/w) and other trace catechin derivatives. It was prepared as a 137.5 mg/kg GTE suspension (lower dose of GTE, lGTE) and a 275 mg/kg GTE suspension (higher dose of GTE, hGTE) in 0.1 mL distilled water. For the EGCG treatment, EGCG was prepared as a 96.25 mg/kg EGCG suspension (lower dose of EGCG, lEGCG) and a 192.5 mg/kg EGCG suspension (higher dose of EGCG, hEGCG) in 0.1 mL distilled water (70% of the GTE doses). Mice were treated with GTE or EGCG once per two days, administrated intragastrically, starting from 5 days prior to EAU induction through d21pi. The treatment strategy was demonstrated in Fig. S15. Equal volume of distilled water or 1 mg/kg dexamethasone (Dex; Sigma-Aldrich Corp., St. Louis, MO, USA.) was administrated as negative and positive controls, respectively. For the GTE treatment, mice were randomly divided into eight groups: (1) mice mock induced with PBS and fed with distilled water (PBS + Water, n = 6); (2) mice received EAU induction and fed with distilled water (EAU + Water, n = 12); (3 and 4) EAU mice with lGTE or hGTE administration (EAU + lGTE, n = 6; EAU + hGTE, n = 12); (5 and 6) 1 mg/kg Dex orally treated animals with EAU induction or PBS mock induction (EAU + Dex, n = 12; PBS + Dex, n = 10); (7 and 8) mice mock induced with PBS and fed with lGTE or hGTE (PBS + lGTE, n = 4; PBS + hGTE, n = 10). Similarly, mice were randomly divided into four groups for the EGCG treatment: (1 and 2) EAU + lEGCG, n = 6; EAU + hEGCG, n = 6; (3 and 4) PBS + lEGCG, n = 4; PBS + hEGCG, n = 4. At d21pi, mice were sacrificed and eyes were collected for histopathological assessment and gene expression studies. Spleen, liver and kidney were collected for histopathological assessment of systemic effects of GTE and EGCG treatments. The experiments were repeated twice.

### Clinical manifestations scoring

Clinical features of intraocular inflammation were assessed using confocal scanning laser ophthalmoscopy (cSLO) imaging, graded on a scale of 0–4, as described previously^24^.

### Evaluation of retinal-choroidal thickness (RCT), major retinal vessel diameter and electroretinography (ERG) amplitudes

To quantify the EAU severity in various perspectives, RCT, major retinal vessel diameter, and visual function were measured by spectral-domain optical coherence tomography (SD-OCT), fundus fluorescein angiography (FFA), and ERG, respectively. The procedures were described previously^6^.

### Histology and spleen quantification

Mice were perfused intracardially with 0.01 M sterile PBS followed by 4% paraformaldehyde in PBS. The eyes, liver, kidney and spleen were further fixed in 10% formalin for 24 hours at 4 °C and embedded in paraffin. 5 μm sections of the tissues were cut through the pupil-optic nerve position and stained with Hematoxylin and Eosin. Spleen was weighed and imaged for size measurement by Image J software (version 1.50i; NIH, USA).

### Quantification of gene expression

Retina was immersed in 350 μl TRIzol reagent (Invitrogen). Total RNA was isolated and treated with RNase-free DNase I (Qiagen) according to the manufacturer’s protocol. RNA was reverse transcribed into cDNA using SuperScript III reverse transcriptase (Invitrogen). Polymerase chain reaction (PCR) was performed using an iCycler PCR instrument (Bio-Rad) and LightCycler 480 II real-time PCR (RT-PCR) (Roche Applied Science). The gene-specific primers for cDNA sequences were listed in Table S1. The thermal cycle was preincubation at 95 °C for 10 min followed by 45 cycles with denaturation at 95 °C for 15 sec, annealing/extension temperature at 60 °C for 1 min. All samples were run in triplicate. The threshold cycle value of the target gene (C_T*target*_) was corrected with that of the internal control gene β-actin (C_T*β-actin*_). The expression values in each gene [2^−(CT*target-*CT*β-actin*)^] were normalized. Fold changes resulting from treatment were obtained by comparing the normalized expression values for each gene in the treatment group and in the non-induced water treatment group.

### Statistical analysis

All data are expressed as mean ± standard error of mean (SEM). SPSS version 19.0 (IBM Corp., Armonk, NY) and GraphPad Prism 5.0 (GraphPad Software, Inc., La Jolla, CA) software were used for the statistical analysis. Nonparametric Mann-Whitney *U* test was used to compare the medians of two groups. Kruskal-Wallis test was employed to compare the differences among EGCG (low and high) and GTE (low and high) treatment groups. To compare the differences before and after treatments in individuals, Wilcoxon Signed-Rank test was performed. All statistical tests were two-tailed and were performed using a significance level of *P* = 0.05.

## Supplementary information

Supplementary data
