# Supplementary material for: Green tea catechins alleviate autoimmune symptoms and visual impairment in a murine model for human chronic intraocular inflammation by inhibiting Th17-associated pro-inflammatory gene expression
Source: Sci Rep. 2019 Feb 19;9:2301. doi: 10.1038/s41598-019-38868-1 (PMC6381204; doi:10.1038/s41598-019-38868-1)
Supplement: Supplementary file 1 — Supplementary data [file 41598_2019_38868_MOESM1_ESM.pdf]

Green tea catechins alleviate autoimmune symptoms and visual impairment in a murine model for human chronic intraocular inflammation by inhibiting Th17-associated pro-inflammatory gene expression

[1,2]Jian Li, MD, PhD, [1]Yolanda Wong Ying Yip, MSc, [3]Jialin Ren, PhD, [4]Wing Ki Hui, MBBS, [1]Jing Na He, MSc, [3]Qiu Xiao Yu, MSc, [1]Kai On Chu, PhD, [1]Tsz Kin Ng, PhD, [3]Sun On Chan, DPhil, [1]Chi Pui Pang, DPhil, [1]Wai Kit Chu, DPhil*

[1]Department of Ophthalmology & Visual Sciences, The Chinese University of Hong Kong, Hong Kong

[2]Department of Ophthalmology, Affiliated Hangzhou First People's Hospital, Zhejiang University School of Medicine, Hangzhou, China

[3]School of Biomedical Sciences, The Chinese University of Hong Kong, Hong Kong

[4]Bachelor of Medicine and Bachelor of Surgery Programme, The Chinese University of Hong Kong

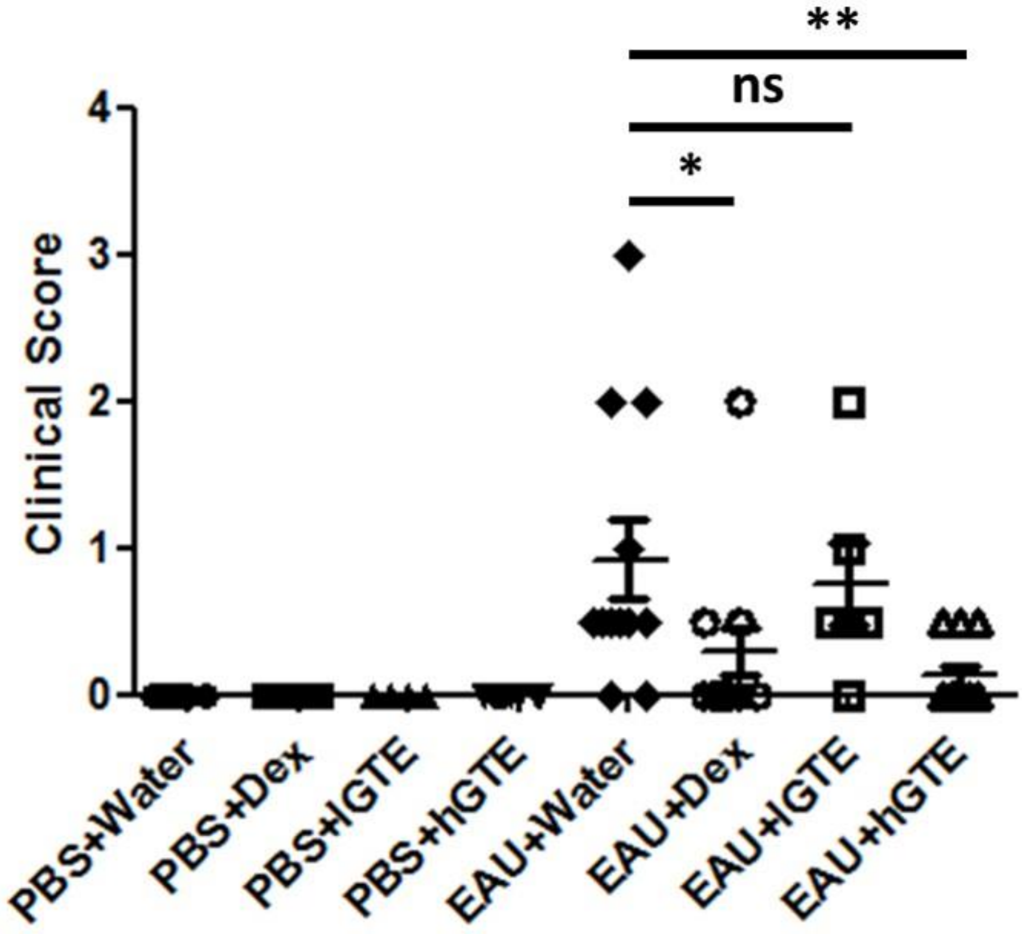

Figure S1. Clinical scores of the inflammatory features. The clinical scores were reduced significantly after Dex and hGTE treatment, but not after lGTE treatment. Individual data points are shown. Data are also presented as mean ± SEM and analyzed by Mann–Whitney *U* test (* $P < 0.05$, ** $P < 0.01$, ns = no significance).

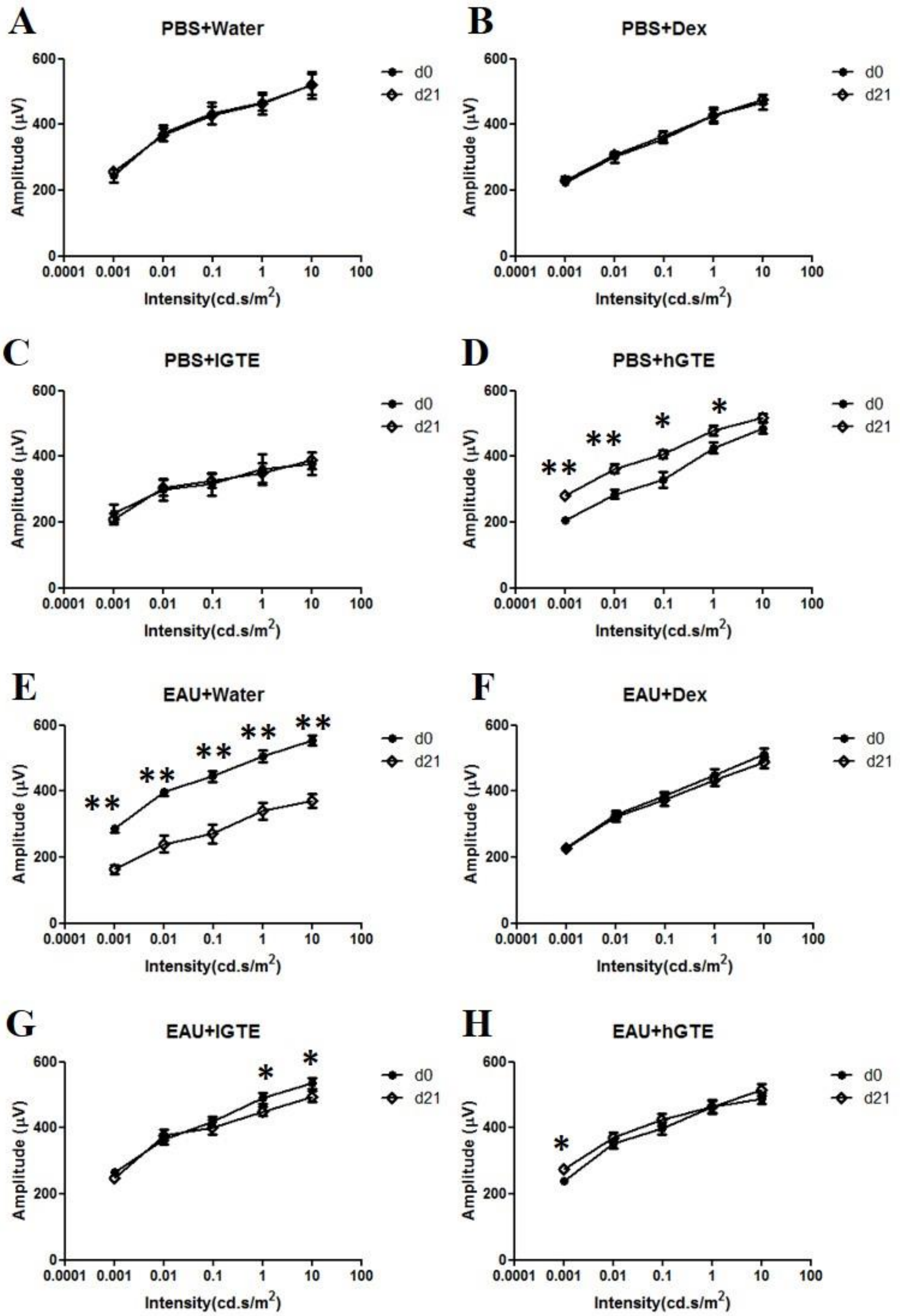

Figure S2. Intra-group comparisons of scotopic ERG amplitudes between baseline (d0) and d21pi. Scotopic amplitudes were not changed significantly during the 21-day experiment in non-induced groups treated with water, Dex and lGTE **(A to C)**. Increases of scotopic ERG

amplitudes were observed in PBS+hGTE **(D)**. Scotopic ERG amplitudes were decreased significantly by EAU induction **(E)**. Scotopic ERG amplitudes in lGTE-treated EAU animals were slightly decreased at d21pi **(G)**. Dex **(F)** and hGTE treatments **(H)** maintained the scotopic ERG amplitudes during the EAU induction. Data are presented as mean ± SEM. Wilcoxon Signed-Rank test was used for paired comparisons (* $P < 0.05$, ** $P < 0.01$).

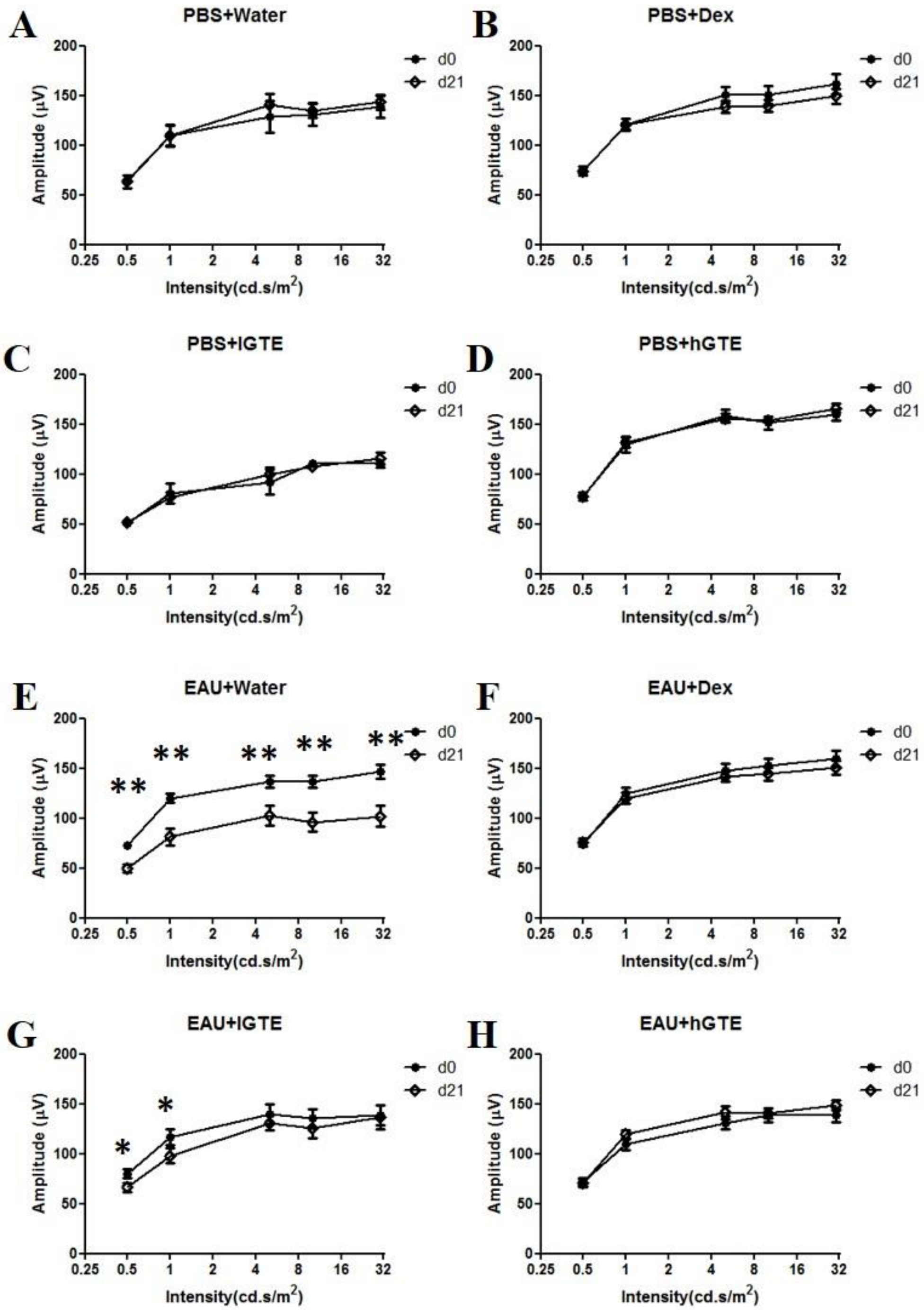

Figure S3. Intra-group comparisons of photopic ERG amplitudes between baseline (d0) and d21pi. Photopic amplitudes were not changed significantly during the 21-day experiment in non-induced groups treated with water, Dex, lGTE and hGTE **(A to D)**. Photopic ERG

amplitudes were decreased significantly by EAU induction **(E)**. Photopic ERG amplitudes in lGTE-treated EAU animals were slightly decreased at d21pi **(G)**. Dex **(F)** and hGTE treatments **(H)** maintained the photopic ERG amplitudes during the EAU induction. Data are presented as mean ± SEM. Wilcoxon Signed-Rank test was used for paired comparisons (* $P < 0.05$, ** $P < 0.01$).

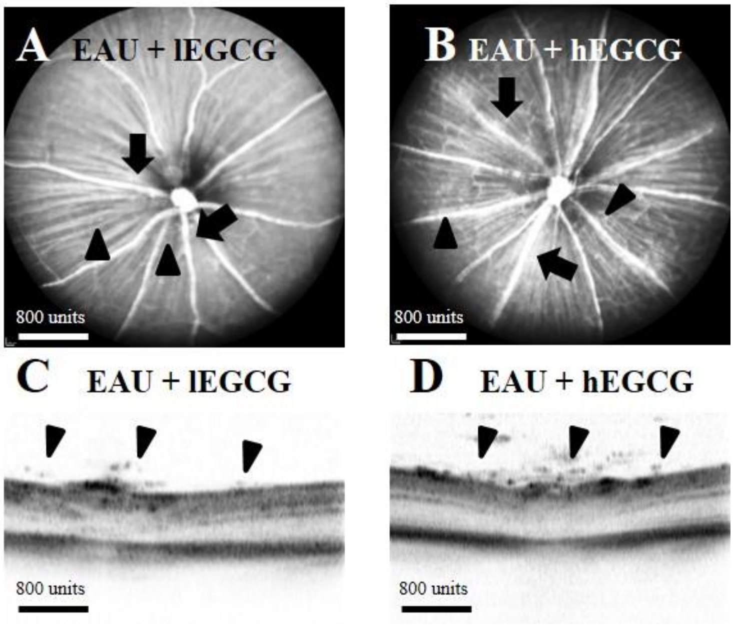

Figure S4. Clinical manifestation of inflammation observed by cSLO (A and B) and SD-OCT (C and D). Infiltrating cells (*arrow heads*) and vasculitis (*arrows*) were observed in EAU animals treated with lEGCG and hEGCG.

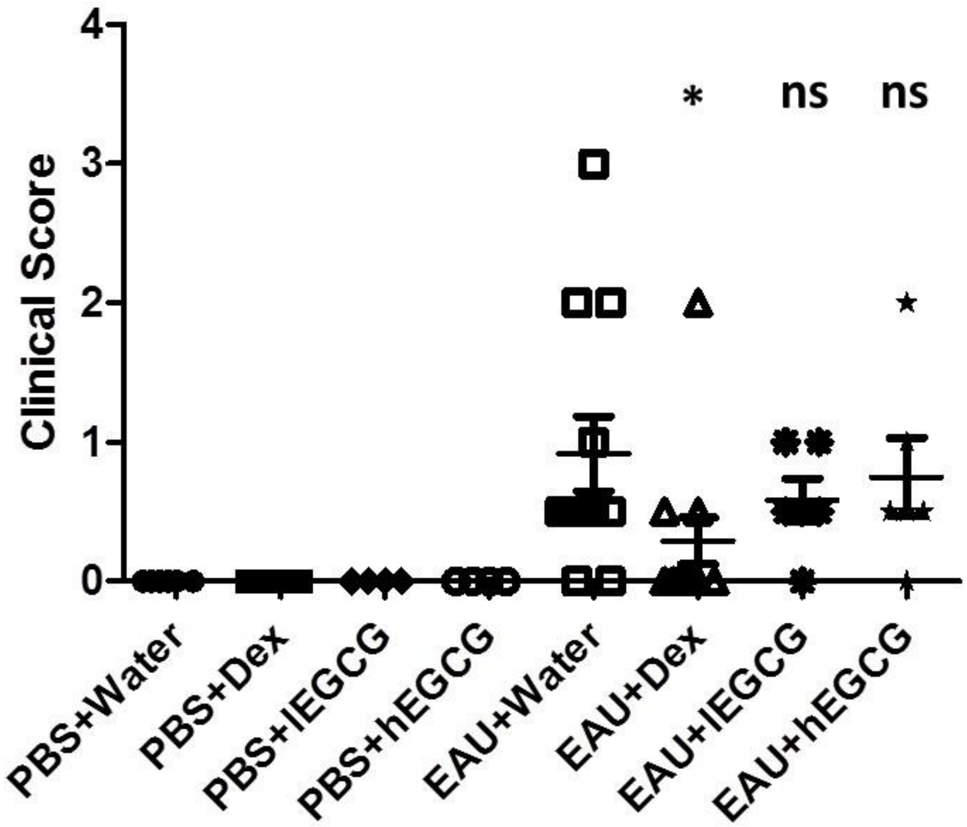

Figure S5. Clinical scores of the inflammatory features. The clinical scores in either lEGCG- or hEGCG-treated EAU group had no difference from EAU animals treated with water. Individual data points are shown. Data are also presented as mean ± SEM and analyzed by Mann–Whitney U test (* $P < 0.05$, ns = no significance).

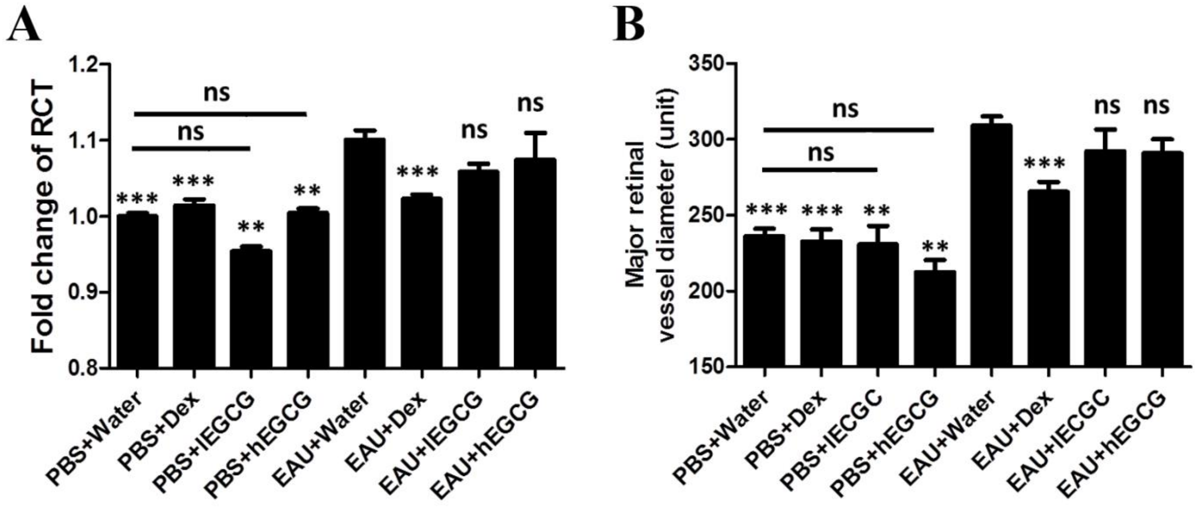

Figure S6. The fold change of RCT (A) and the major retinal vessel diameter (B) assessments. The asterisk marked above each bar represents the statistical significance of difference between EAU+Water group and the corresponding group. Data are presented as mean ± SEM and analyzed by Mann–Whitney *U* test (*** $P < 0.001$, ** $P < 0.01$, ns = no significance).

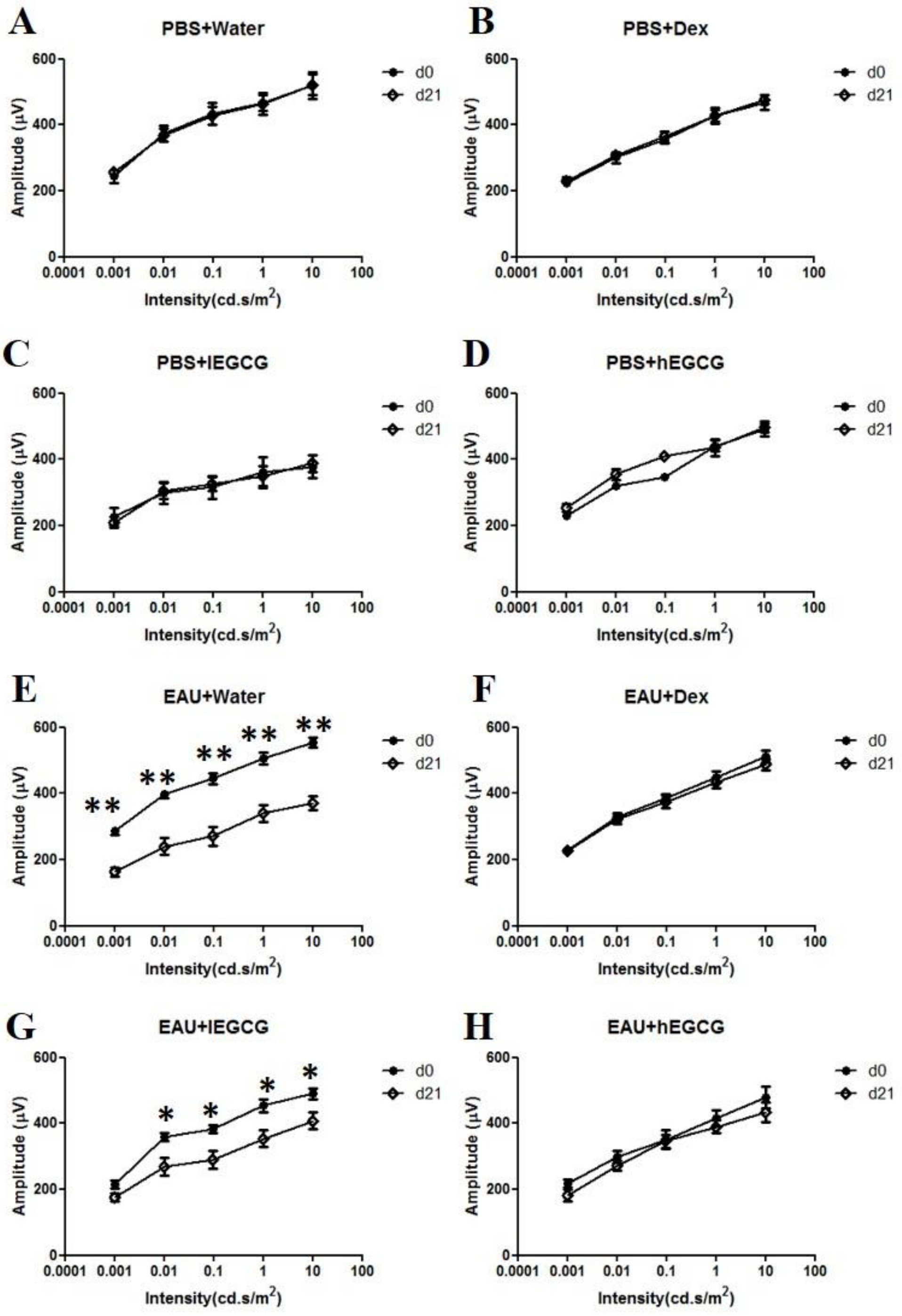

Figure S7. Intra-group comparisons of scotopic ERG amplitudes between baseline (d0) and d21pi. Scotopic amplitudes were not changed significantly during the 21-day experiment in non-induced groups treated with water, Dex and two doses of EGCG **(A to D)**. Scotopic ERG

amplitudes were decreased significantly by EAU induction **(E)**. Scotopic ERG amplitudes in lEGCG-treated EAU animals were also decreased significantly on d21pi **(G)**. Dex **(F)** and hEGCG treatments **(H)** maintained the scotopic ERG amplitudes during EAU. Data are presented as mean ± SEM. Wilcoxon Signed-Rank test was used for paired comparisons (* $P < 0.05$, ** $P < 0.01$).

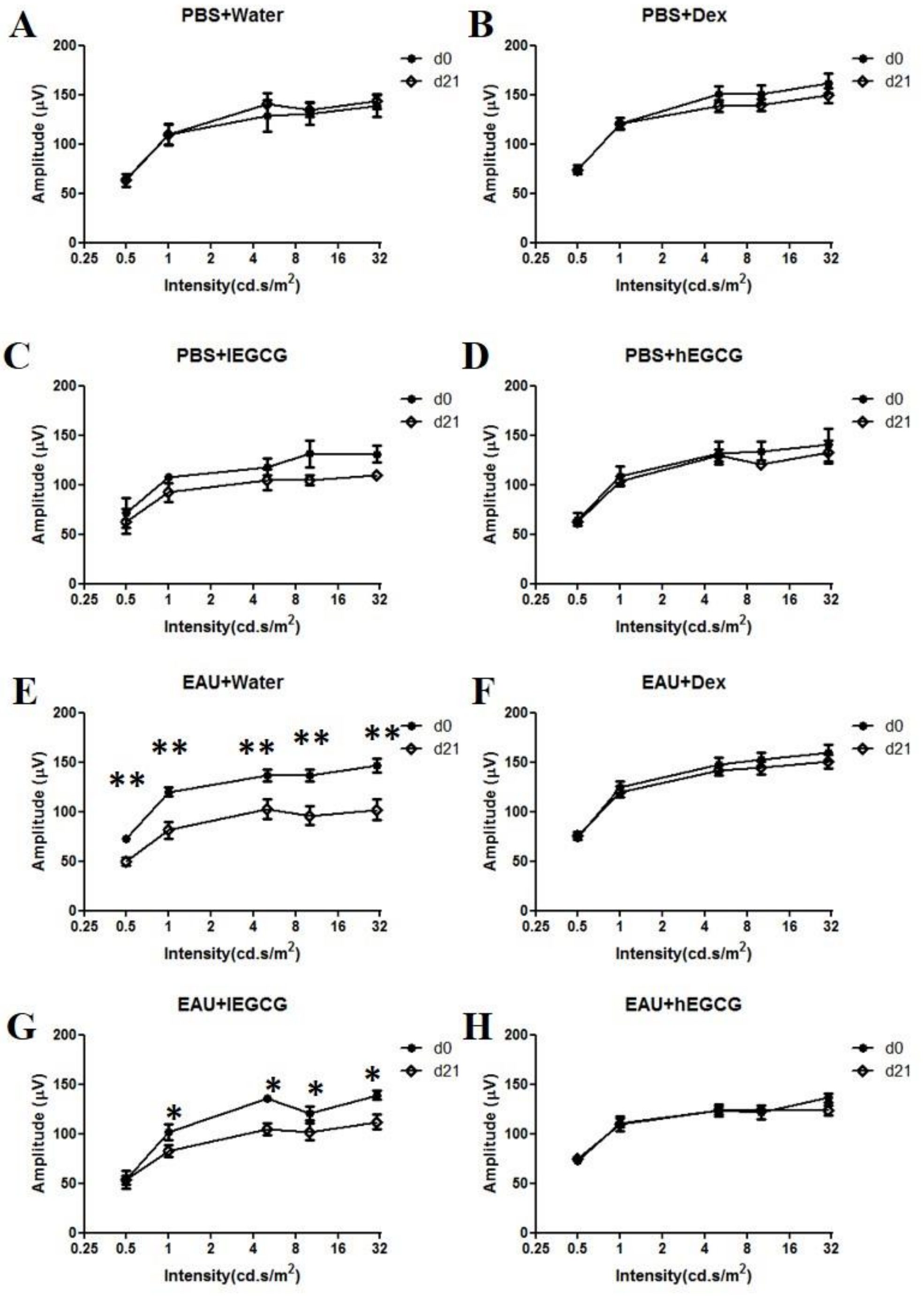

Figure S8. Intra-group comparisons of photopic ERG amplitudes between baseline (d0) and d21pi. Photopic amplitudes were not changed significantly during the 21-day experiment in non-induced groups treated with water, Dex, lEGCG, and hEGCG **(A to D)**. Photopic ERG amplitudes were decreased significantly by EAU induction **(E)**. Photopic ERG amplitudes in

lEGCG-treated EAU animals were decreased significantly on d21pi **(G)**. Dex **(F)** and hEGCG treatments **(H)** maintained the photopic ERG amplitudes during EAU disease. Data are presented as mean ± SEM. Wilcoxon Signed-Rank test was used for paired comparisons (* $P < 0.05$, ** $P < 0.01$).

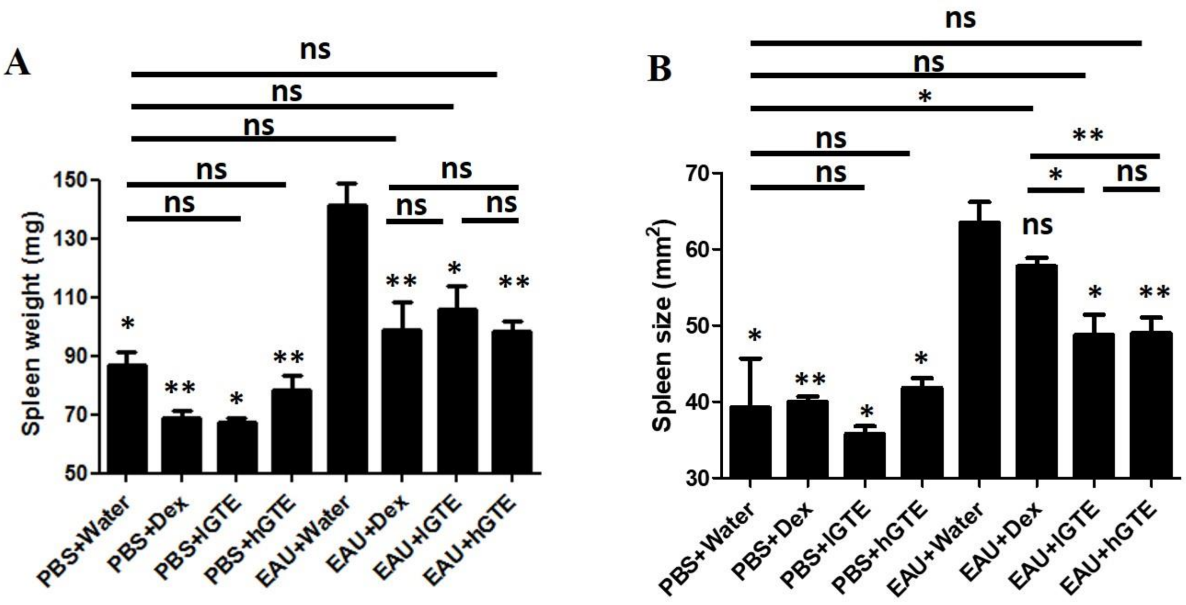

Figure S9. Assessments of the spleen weight and spleen size after GTE treatments on d21pi. Spleen weight was increased significantly after EAU induction. The increased spleen weight caused by EAU was alleviated by Dex, lGTE, or hGTE treatment **(A)**. Spleen size was increased significantly by EAU induction. The enlarged spleen size was attenuated by lGTE and hGTE, but not Dex **(B)**. The asterisk marked above each bar represents the statistical significance of comparison between EAU+Water group and the corresponding group. Data are presented as mean ± SEM and analyzed by Mann–Whitney *U* test (* $P < 0.05$, ** $P < 0.01$, ns = no significance).

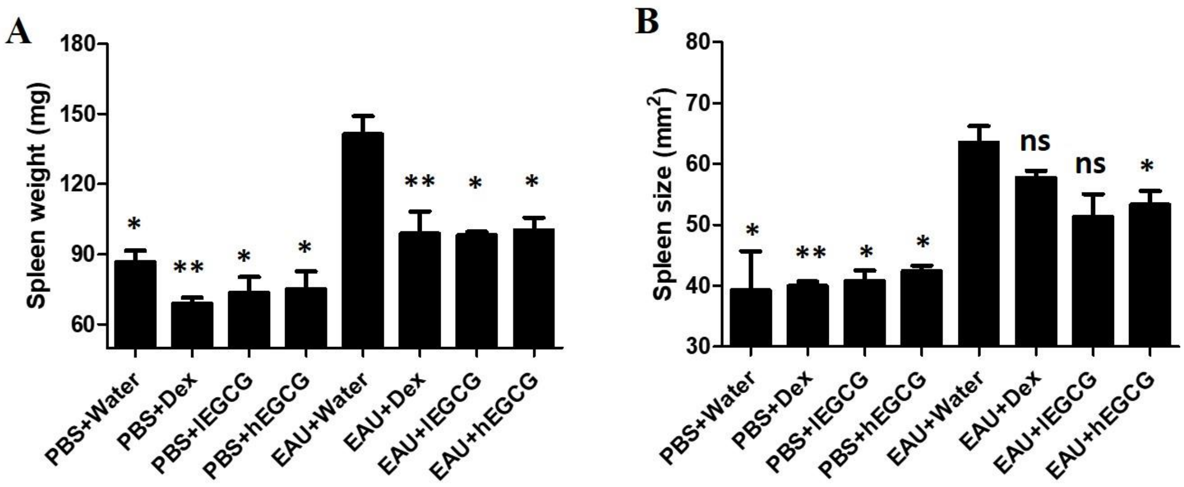

Figure S10. Assessments of the spleen weight and spleen size after EGCG treatments on d21pi. Spleen weight was increased significantly after EAU induction. The increased spleen weight caused by EAU was alleviated by Dex, lEGCG, or hEGCG treatment **(A)**. Spleen size was increased significantly by EAU induction. The enlarged spleen size was attenuated by hEGCG, but not lEGCG and Dex **(B)**. The asterisk marked above each bar represents the statistical significance of comparison between EAU+Water group and the corresponding group. Data are presented as mean ± SEM and analyzed by Mann–Whitney *U* test (* $P < 0.05$, ** $P < 0.01$, ns = no significance).

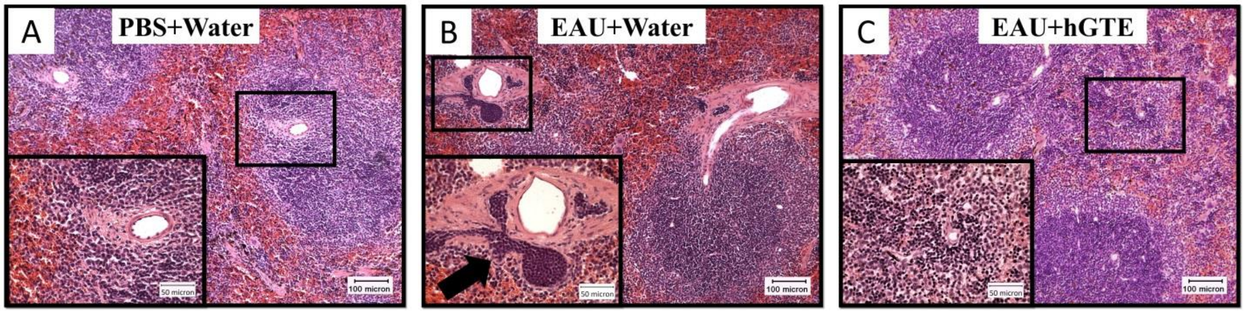

Figure S11. Histological observations of spleen on d21pi. No detectable defect was observed in spleen of PBS+Water group **(A)**. Accumulated cells (*arrow*) were observed in spleen of EAU+Water group **(B)**. hGTE treatment alleviated the cell infiltration in EAU mice **(C)**. Selected areas are enlarged and shown on the bottom left corner.

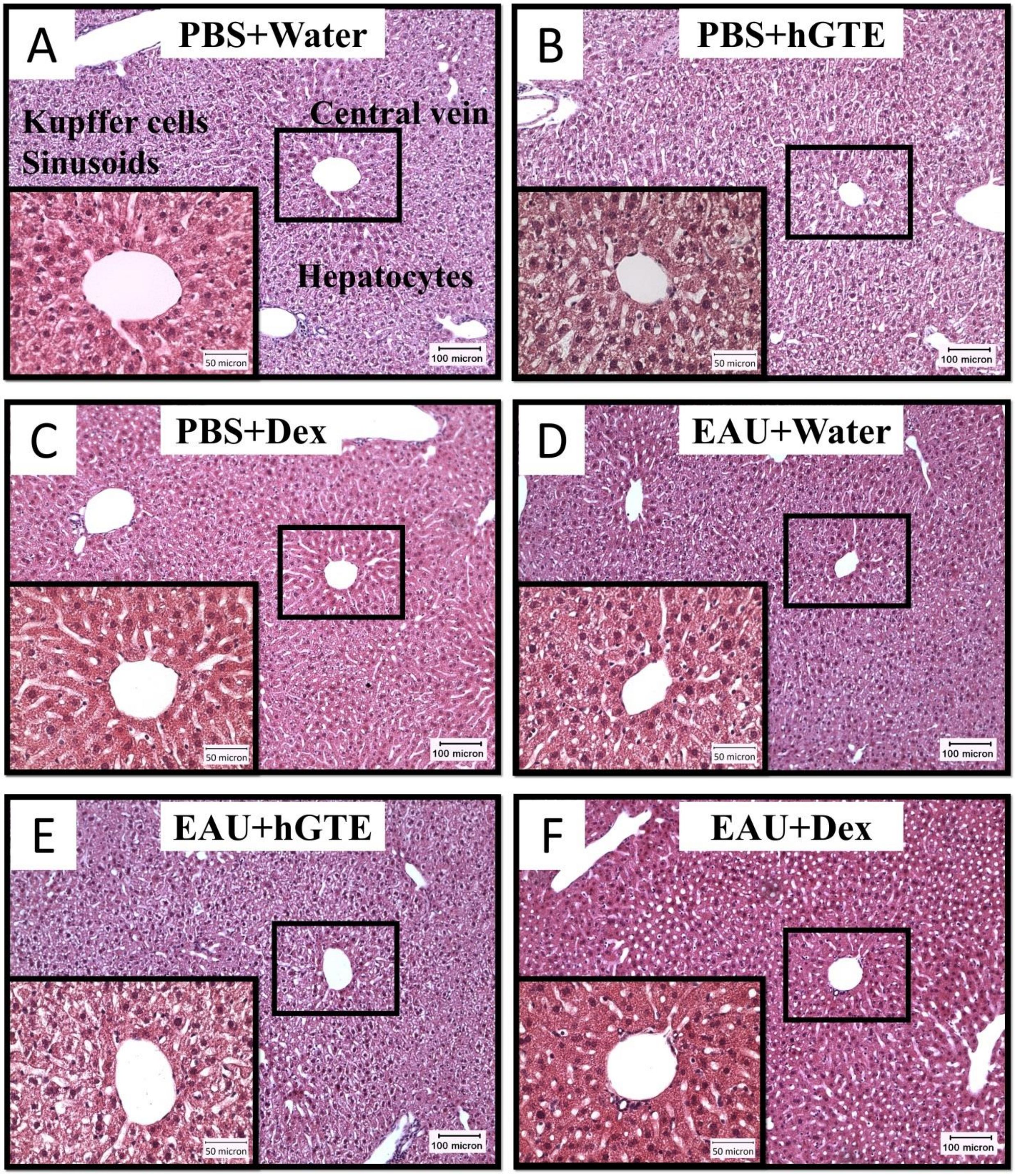

Figure S12. Histological observations of liver on d21pi. No detectable defect was observed in liver of non-induced animals fed with water, hGTE, and Dex **(A to C)**. No detectable damage in

liver was observed in EAU animals treated with water, hGTE, and Dex **(D to F)**. Selected areas are enlarged and shown on the bottom left corner.

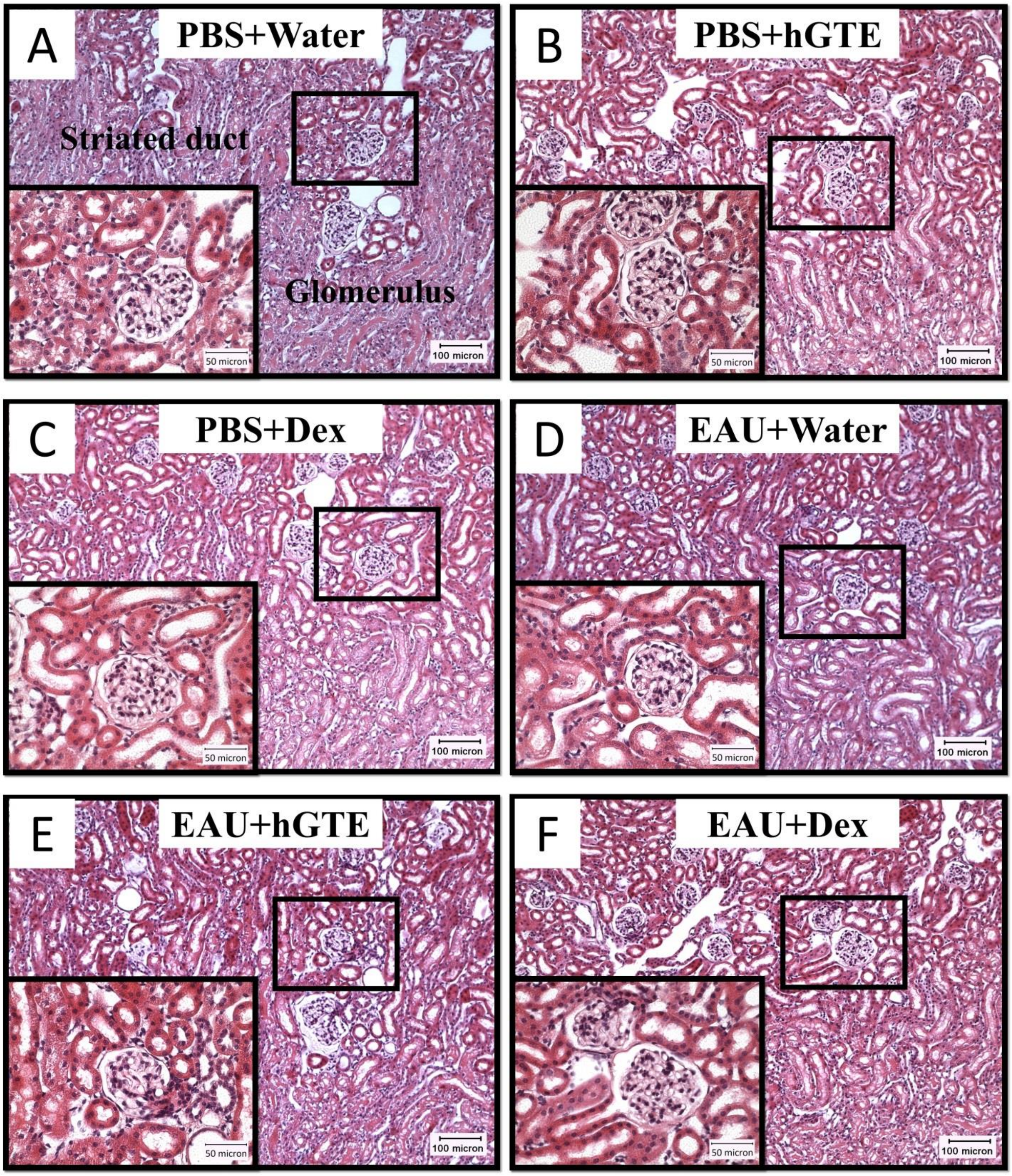

Figure S13. Histological observations of kidney on d21pi. No detectable defect was observed in kidney of non-induced animals fed with water, hGTE, and Dex **(A to C)**. No detectable

damage in kidney was observed in EAU animals treated with water, hGTE, and Dex **(D to F)**.

Selected areas are enlarged and shown on the bottom left corner.

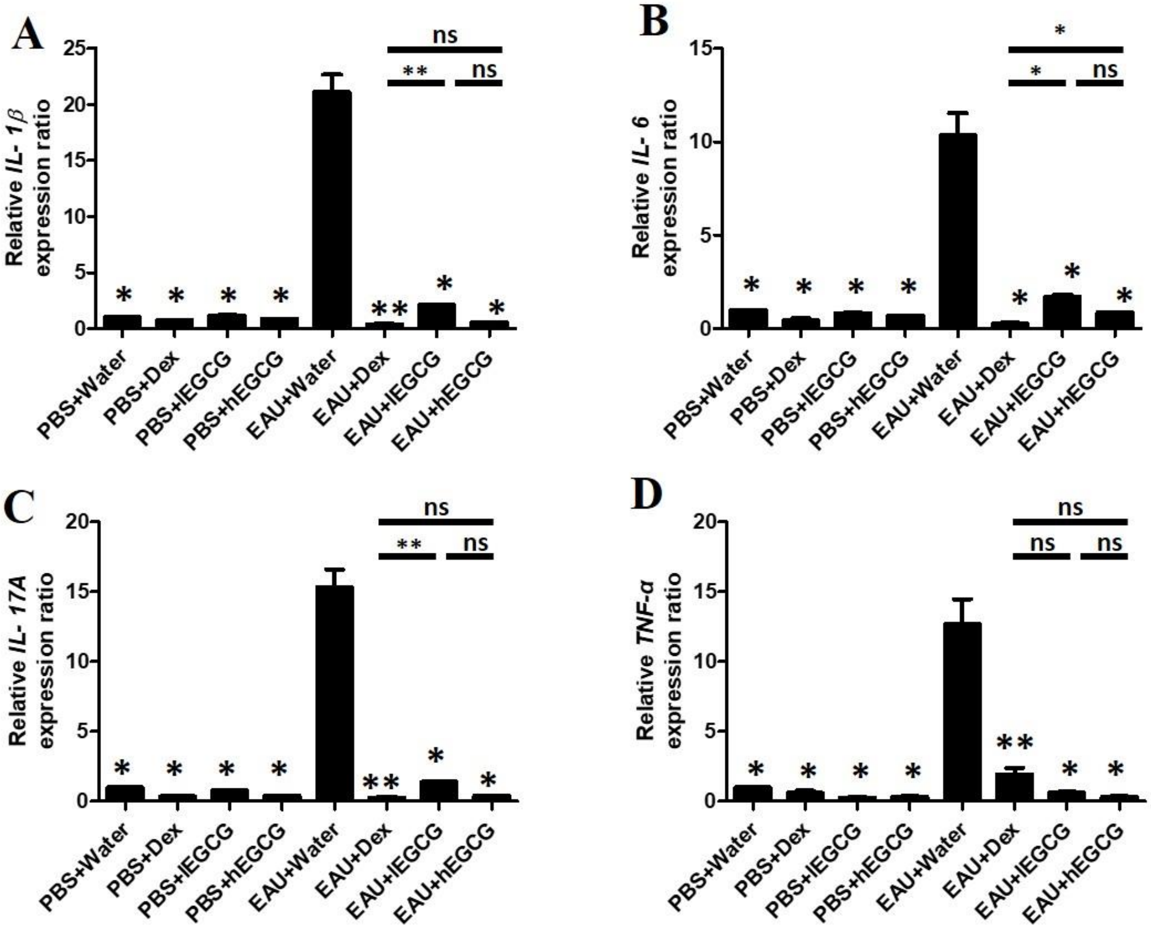

Figure S14. Effects of EGCG on *IL-1β, IL-6, IL-17A,* and *TNF-α* mRNA expression in EAU retina. The asterisk marked above each bar represents the statistical significance of comparison between EAU+Water group and the corresponding group. Data are shown as mean ± SEM and analyzed using Mann-Whitney *U* test (* $P < 0.05$, ** $P < 0.01$, ns = no significance).

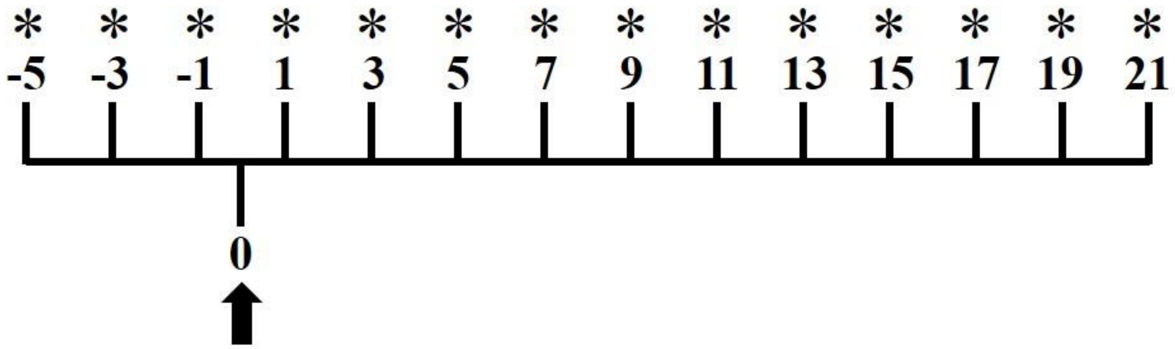

Figure S15. Demonstration of the treatment strategy. Mice were treated with controls, lower dose or higher dose of GTE or EGCG once per two days (asterisks) starting from 5 days prior to EAU induction (arrow) until day 21 postimmunization.

Table S1

Gene-specific primers for mouse *TNF-α*, *IL-1β*, *IL-6*, *IL-17A*, and *β-actin*.

Tumor necrosis factor alpha (*TNF-α*)	
Forward	5’- GCC ACC ACG CTC TTC TGT CT -3’
Reverse	5’- GGT CTG GGC CAT AGA ACT GAT G -3’
Interleukin-1 beta (*IL-1β*)	
Forward	5’- CAG CTC ATA TGG GTC CGA CA -3’
Reverse	5’- CTG TGT CTT TCC CGT GGA CC -3’
Interleukin-6 (*IL-6*)	
Forward	5’- CTG CAA GAG ACT TCC ATC CAG -3’
Reverse	5’- AGT GGT ATA GAC AGG TCT GTT GG -3’
Interleukin-17A (*IL-17A*)	
Forward	5’- AAG GCA GCA GCG ATC ATC C -3’
Reverse	5’- GGA ACG GTT GAG GTA GTC TGA G -3’
Beta-actin (*β-actin*)	
Forward	5’- TGT TAC CAA CTG GGA CGA CA -3’
Reverse	5’- GGG GTG TTG AAG GTC TCA AA -3’
